# Modifying Carbohydrate Supply to Fruit during Development Changes the Composition and Flavour of *Actinidia chinensis* var. *chinensis* ‘Zesy002’ Kiwifruit

**DOI:** 10.3390/plants10071328

**Published:** 2021-06-29

**Authors:** Danielle Le Lievre, Rachelle Anderson, Helen Boldingh, Janine Cooney, Richard Seelye, Nick Gould, Denise Hunter, Dwayne Jensen, Trisha Pereira, Mark Wohlers, Mike Clearwater, Annette Richardson

**Affiliations:** 1 School of Science, University of Waikato, Private Bag 3105, Hamilton 3240, New Zealand; danielle.lelievre@waikato.ac.nz (D.L.L.); mike.clearwater@waikato.ac.nz (M.C.); 2 Te Puke Research Centre, The New Zealand Institute for Plant & Food Research Limited (PFR), 412 No. 1 Road, RD2, Te Puke 3182, New Zealand; rachelle.anderson@plantandfood.co.nz (R.A.); nick.gould@plantandfood.co.nz (N.G.); 3 PFR, Ruakura Research Centre, Private Bag 3105, Hamilton 3240, New Zealand; helen.boldingh@plantandfood.co.nz (H.B.); janine.cooney@plantandfood.co.nz (J.C.); richard.seelye@plantandfood.co.nz (R.S.); dwayne.jensen@plantandfood.co.nz (D.J.); trisha.pereira@plantandfood.co.nz (T.P.); 4 PFR, Mt Albert Research Centre, Private Bag 92169, Auckland 1142, New Zealand; denise.hunter@plantandfood.co.nz (D.H.); Mark.Wohlers@plantandfood.co.nz (M.W.); 5 PFR, Kerikeri Research Centre, 121 Keri Downs Road, RD1, Kerikeri 0294, New Zealand

**Keywords:** acid, cytokinin, girdling, kiwifruit, leaf to fruit ratio, consumer, starch, sugar

## Abstract

Consumer acceptance of fruit is determined by size, flavour and ripeness. In this study we investigated how altering the carbohydrate supply to *Actinidia chinensis* var. *chinensis* ‘Zesy002’ kiwifruit altered the balance between growth and accumulation of metabolites. Canes were phloem girdled and fruit thinned to a leaf-to-fruit ratio (L:F) of either 2 (Low carbohydrate) or 6 (High carbohydrate) at either 38 (Early) or 86 (Late) days after anthesis (DAA) and compared with ungirdled control canes with a L:F of 3. Fruit growth, metabolite accumulation, cytokinin concentrations and maturation were monitored and the sensory attributes of ripe fruit were assessed. The final weight of Early-High and Late-High carbohydrate fruit was 38% and 16% greater compared with control fruit. High carbohydrate fruit had increased starch, soluble sugar and cytokinin concentrations and fruit began to mature earlier and those with a Low carbohydrate had decreased concentrations and matured later compared with control fruit. Control fruit were described by consumers as more acidic and under-ripe compared with those from Early-High carbohydrate canes, but as sweeter than those from Low carbohydrate canes. This study showed that carbohydrate supply can have a major impact on the growth, sugar accumulation and maturity of ‘Zesy002’ fruit sinks.

## 1. Introduction

The yellow-fleshed kiwifruit cultivar *Actinidia chinensis* (Planch.) var. *chinensis* ‘Zesy002’ (marketed as Zespri SunGold™ Kiwifruit) is relatively new to the marketplace. It replaced the first globally released yellow-fleshed cultivar ‘Hort16A’ which was decimated when the disease *Pseudomonas syringae* pv. *actinidiae* biovar 3 was introduced to New Zealand [1]. ‘Zesy002’is a cultivar that can produce high yields of very large fruit [2]. In many fruit crops, including most kiwifruit cultivars, there are generally negative relationships between increasing fruit number and fruit size, and between fruit size and fruit dry matter content (DM) [3,4,5,6,7]. In contrast, smaller ‘Zesy002’ fruit tend to have lower DM [8] and hence are less appealing to consumers [9].

Vine carbohydrate partitioning must be well managed to optimise the size and flavour of fruit for consumers [10]. For kiwifruit, the sugar and acid balance is a key indicator of fruit flavor [11,12] and can be predicted from the DM of unripe fruit [13,14]. Previous studies of kiwifruit have also shown that the ripeness of fruit also has a large impact on the flavour and texture of fruit and hence consumer responses [15,16].

The development of kiwifruit can be divided into two main stages, rapid fruit growth (0–60 days after antheisis (DAA)) in green-fleshed ‘Hayward’ (*A. chinensis* var. *deliciosa*) and ‘Hort16A’) [17,18] when cell division and rapid cell enlargement occurs [19] and a second period of slower growth (until 140 DAA in ‘Hayward’ and ‘Hort16A’) [18,20] when cells continue to enlarge, rapid accumulation of dry matter occurs and fruit mature [21]. During the initial stage of rapid fruit growth, starch concentration declines [4] and both glucose and quinic acid accumulate in developing fruit [18,22]. As fruit growth slows, during the second stage of development starch accumulates rapidly in fruit [23,24], quinic acid concentrations decline and citric acid concentrations increase [25]. Later, fruit growth stops and fruit maturation begins with the breakdown of starch, an increase of soluble sugars, a gradual losss in firmness, changes in fruit pigments and the accumulation of volatile metabolites [18,26,27,28]. While the growth and development of several kiwifruit cultivars has been described (‘Hayward’ [29], ‘Hort16A’ [18], *Actinidia arguta* genotypes [30]), the growth and development of ‘Zesy002’ fruit has not yet been described.

Studies of kiwifruit have shown that factors that affect carbohydrate source supply to fruit sinks can influence their final growth and composition. These factors can include crop load [3], leaf-to-fruit (L:F) ratio [31,32], girdling [33], application of growth regulators [8,34], pollination [35], application of nitrogen fertiliser [36], light [37] and temperature [38]. Girdling (to prevent carbohydrate moving to nonfruit sinks e.g., roots) and thinning studies (to increase the leaf:fruit ratio) of ‘Hayward’ vines have shown that effects of changing carbohydrate source supply on fruit size generally occur during rapid fruit growth while effects on fruit dry matter accumulation are seen during the second stage of fruit growth [39,40]. Applications of the synthetic cytokinin *N*-(2-chloro-4-pyridyl)-*N*′-phenylurea (CPPU) during rapid early fruit growth have suggested that increased fruit size is due to increased cell size [41] through greater water uptake to balance higher glucose and fructose concentrations in fruit [40]. A recent study of a red *A. chinensis* var. *chinensis* genotype has shown that cytokinin biosynthetic genes involved in cell expansion in fruit were down-regulated when the carbohydrate supply to fruit was reduced from 28 days after anthesis [42]. Effects of carbohydrate supply on fruit dry matter accumulation have not been investigated but could be similar to changes in carbohydrate metabolism shown in genotypes with contrasting DM [21]. The changes in kiwifruit composition due to changing the amount of carbohydrate supplied at different stages of development has not previously been described.

Carbohydrate source supply can influence the maturation of apple [43], peach [44], cherry [45] and kiwifruit [35,37] as measured by changes in soluble solids content (SSC), colour and firmness. Increases in SSC in fruit have been related to increases in soluble sugar accumulation in kiwifruit vines with a low crop load [13] or in girdled vines [46]. Studies have suggested that the start of fruit softening is associated with the cessation of fruit growth and carbohydrate accumulation [47]. Fruit colour development in gold and red kiwifruit is generally related to the decline in chlorophyll pigments rather than the accumulation of carotenoids [26] and anthocyanins [48] although carbohydrate supply has been shown to influence accumulation of anthocyanins in red-fleshed fruit [49] However, no fruit developmental studies have shown how altering the fruit carbohydrate supply influences fruit maturation of kiwifruit.

In the current study we investigated how manipulating the supply of source carbohydrate at different stages of fruit sink development influenced the growth and composition of ‘Zesy002’ kiwifruit. Treatments included Low (one to two leaves per fruit) or High (six leaves per fruit) carbohydrate supply, that were applied to individual canes together with a basal phloem girdle either Early (38 DAA) or Late (86 DAA) in fruit development. The aim of this work was to provide greater understanding of how changing carbohydrate supply at different stages of fruit development affected fruit growth, endogenous cytokinin concentrations, accumulation of key flavour metabolites and maturation of fruit. We also wanted to determine whether these changes affected consumer preferences for fruit. Our hypothesis was that altering the carbohydrate source supply to fruit sinks at key stages of rapid fruit growth or starch accumulation changes the balance between fruit growth and the accumulation of flavour compounds in ‘Zesy002’ fruit and hence their appeal to consumers.

## 2. Results

### 2.1. The Effect of Carbohydrate Supply on Fruit Growth

**Figure 1:**
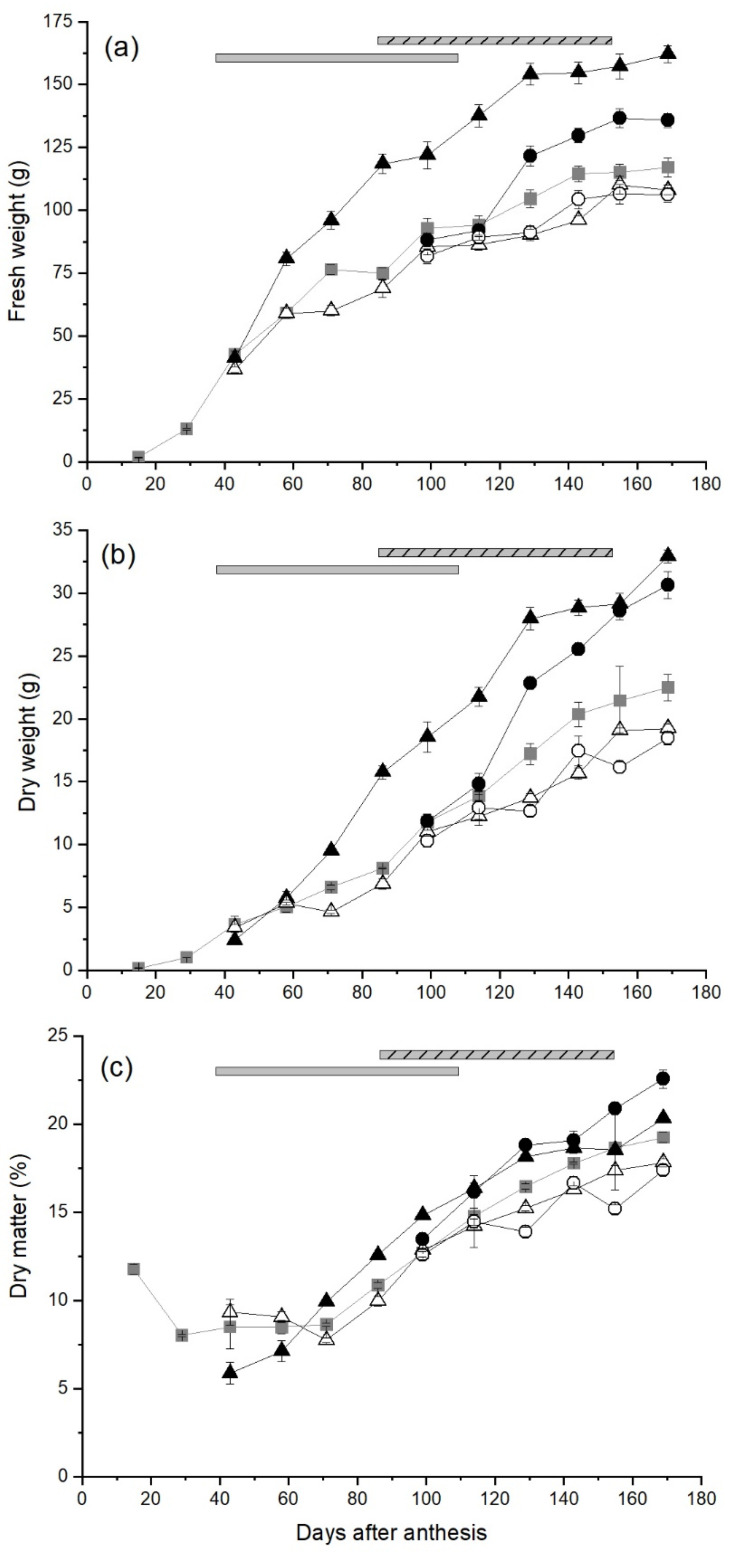
The effect of time of manipulation of carbohydrate supply together with phloem girdling on fruit (**a**) fresh weight (**b**) dry weight and (**c**) dry matter content of *Actinidia chinensis* var. *chinensis* ‘Zesy002’. kiwifruit from anthesis through to harvest. Data are shown for ungirdled control canes (■), early girdled canes with high (▲) or low (△) carbohydrate supply or late girdled canes with high (●) or low (○) carbohydrate supply. Early treatments were applied at 38 days after anthesis with the duration of the open girdle shown by the grey solid bar at the top of each panel. Late treatments were applied at 86 days after anthesis with the duration of the open girdle shown by the grey/black stripped bar at the top of each panel. High carbohydrate supply treatments had six leaves per fruit, while low carbohydrate supply treatments had one or two leaves per fruit. Data are means ± the standard error of the mean (SEM), *n* = 5.

Fruit from ungirdled control canes grew rapidly during the early stages of development, with average fruit FW increasing from 1.69 g at 15 days after anthesis (DAA) to 76.4 g at 71 DAA (Figure 1a). There was then a short plateau in fruit growth until 86 DAA after which growth continued at a steady rate until 143 DAA. Thereafter fruit growth was minimal and fruit from ungirdled control canes weighed 117 g at the final harvest (169 DAA). On canes with the Early-High carbohydrate supply treatment, fruit fresh weight (FW) increased rapidly from soon after treatments were applied (38 DAA) and fruit were significantly (*p* < 0.05 for this and all subsequent statistical comparisons) larger than fruit from all other treatments throughout fruit development, with the average FW reaching 162 g at harvest. Late-High carbohydrate supply treatments applied at 86 DAA stimulated late season fruit growth with fruit reaching 136 g at 169 DAA, significantly larger than fruit from ungirdled control canes. The growth of fruit on canes that had a low carbohydrate supply from early in the season (Early-Low) was significantly less than that of control fruit at 71 DAA, due to an early pause in growth from 58–71 DAA, but the growth of these fruit followed the pattern of control fruit for the rest of the season reaching 108 g at harvest. Average fruit FW of the Late-Low carbohydrate treatment slowed immediately after the treatment was applied but then resumed a similar growth pattern to that of control fruit with a FW of 106 g at harvest.

The average dry weight (DW) of fruit from ungirdled control canes increased steadily during the rapid stage of fresh weight growth to reach 8.13 g at 86 DAA, and then fruit DW increased more rapidly with control fruit weighing 22.5 g DW at harvest (Figure 1b). The stimulation of FW growth in fruit from the Early-High treatment was reflected in their DW growth and fruit from this treatment had significantly higher fruit DW than control fruit from 71 DAA until they reached 32.9 g DW at harvest. There was also a significant increase in the DW growth of fruit from Late-High carbohydrate supply canes compared with that of control fruit from 129 DAA and Late-High fruit reached 30.6 g DW at harvest. The DW of fruit from the Early-Low carbohydrate supply canes generally followed the pattern of the control fruit but with significant delays in DW growth at 71, 129 and 143 DAA. In contrast the DW growth of fruit from canes with the Late-Low carbohydrate supply was significantly lower than of fruit from the ungirdled control canes from 114 DAA and fruit had only reached 18.5 g DW at harvest.

As the FW of fruit increased during the early stages of growth, the DM of control fruit decreased to a minimum of 7.6–8.6% at 43–71 DAA (Figure 1c). However once the period of rapid fruit FW growth had finished, there was a steady increase in the DM of control fruit until harvest when fruit reached 19.2% DM. The DM of fruit from the Early- High treatment increased steadily once treatments were applied and was significantly higher than that of control fruit at 99 and 129 DAA, but not at harvest. 

In contrast the DM content of fruit from the Late-High treatment was significantly higher than that of control fruit from 114 DAA until harvest when Late-High fruit attained 22.6% DM. The DM content of fruit from Late-Low carbohydrate supply canes was significantly lower than that of control fruit from 129 DAA and only reached 17.4% DM at harvest.

### 2.2. Starch and Sugar Concentrations in Fruit during Development

**Figure 2:**
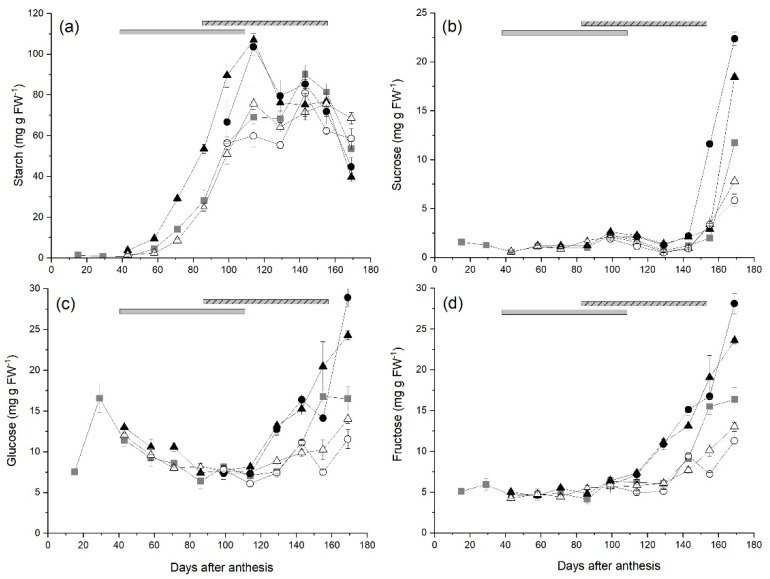
The effect of time of manipulation of carbohydrate supply together with phloem girdling on (**a**) starch (**b**) sucrose (**c**) glucose and (**d**) fructose concentration of *Actinidia chinensis* var. *chinensis* ‘Zesy002’, kiwifruit from anthesis through to harvest. Data are shown for ungirdled control canes (■), early girdled canes with high (▲) or low (△) carbohydrate supply or late girdled canes with high (●) or low (○) carbohydrate supply. Early treatments were applied at 38 days after anthesis with the duration of the open girdle shown by the grey solid bar at the top of each panel. Late treatments were applied at 86 days after anthesis with the duration of the open girdle shown by the grey/black stripped bar at the top of each panel. High carbohydrate supply treatments had six leaves per fruit, while low carbohydrate supply treatments had one or two leaves per fruit. Data are means ± SEM, *n* = 5.

The average starch concentrations in fruit from ungirdled control canes remained low until 58 DAA (4.45 mg g FW^−1^), then accumulated rapidly reaching a plateau at 114–129 DAA before peaking at 90.1 mg g FW^−1^ at 143 DAA (Figure 2a). As control fruit began to mature, their starch concentrations decreased to 53.7 mg g FW^−1^ at harvest (169 DAA). Starch concentrations in fruit from canes with the Early-High carbohydrate supply increased rapidly after the treatment was applied (38 DAA) and were significantly higher than those in fruit from ungirdled control canes from 71 DAA until starch concentrations peaked at 106.9 mg g FW^−1^ at 114 DAA, when cane girdles had healed. The starch concentrations in fruit from canes with the Early-High carbohydrate supply declined from 114–129 DAA, plateaued until 155 DAA, and then decreased to 39.6 mg g FW^−1^ at harvest. There was no effect of the other treatments on the starch concentrations of fruit.

Sucrose concentrations in fruit from ungirdled control canes remained low (<2.0 mg g FW^−1^) until 155 DAA and then increased rapidly to 11.7 mg g FW^−1^ at harvest (Figure 2b) as fruit began to mature. The concentrations of sucrose in fruit from other treatments were similar to those of control fruit until fruit maturation began. From 99 DAA the sucrose concentrations in fruit from canes with the Early-High carbohydrate supply were significantly higher than those in fruit from ungirdled control canes and reached 18.4 mg g FW^−1^ at harvest. The increase in sucrose concentrations in maturing fruit from the Late High treatment began earlier (143 DAA) and concentrations were significantly higher than those in fruit from control canes at 155 DAA and harvest (22.4 mg g FW^−1^). In contrast, the sucrose concentrations in fruit from canes with either the Early-Low (7.79 mg g FW^−1^) or Late-Low (5.85 mg g FW^−1^) were significantly lower than those in control fruit at harvest.

In fruit from ungirdled control canes, the glucose concentrations increased to an early peak of 16.6 mg g FW^−1^ near the beginning of rapid fruit growth (29 DAA), then decreased to concentrations of between 6.38–8.12 mg g FW^−1^ from 86–129 DAA (Figure 2c). As fruit began to mature, glucose concentrations in control fruit increased to a peak of 16.5 mg g FW^−1^ at 155 DAA. There were no significant effects of carbohydrate supply treatments on glucose concentrations in fruit until 129 DAA when glucose concentrations in fruit from both the Early-High and Late-High treatments increased rapidly and were significantly higher than those in control fruit, reaching 24.3 and 28.9 mg g FW^−1^ respectively at harvest. The glucose concentration in fruit from the Early-Low and Late-Low treatments increased at a slower rate than those in control fruit during fruit maturation and at harvest concentrations in fruit from the Late-Low treatment (11.5 mg g FW^−1^) were significantly lower than those in control fruit.

Fructose concentrations in fruit from ungirdled control canes remained between 4.11–6.19 mg g FW^−1^ until 129 DAA. As fruit matured, fructose concentrations in control fruit increased steadily to a peak of 16.4 mg g FW^−1^ at harvest (Figure 2d). In a manner similar to glucose, there were no significant effects of carbohydrate supply treatments on fructose concentrations in fruit until 129 DAA when fruit maturation began. Fructose concentrations in fruit from the Early-High and Late-High treatments were significantly higher than those in control fruit at 129 DAA and concentrations increased rapidly to 23.6 and 28.1 mg g FW^−1^ respectively at harvest, significantly higher than those in control fruit. The increase in fructose concentrations in fruit from canes with both the Early-Low and Late-Low carbohydrate supplies was significantly slower than that in control fruit and at harvest concentrations in fruit from the Late-Low treatment (11.3 mg g FW^−1^) were significantly lower than those in control fruit.

### 2.3. Changes in Organic Acid Concentrations in Fruit

**Figure 3:**
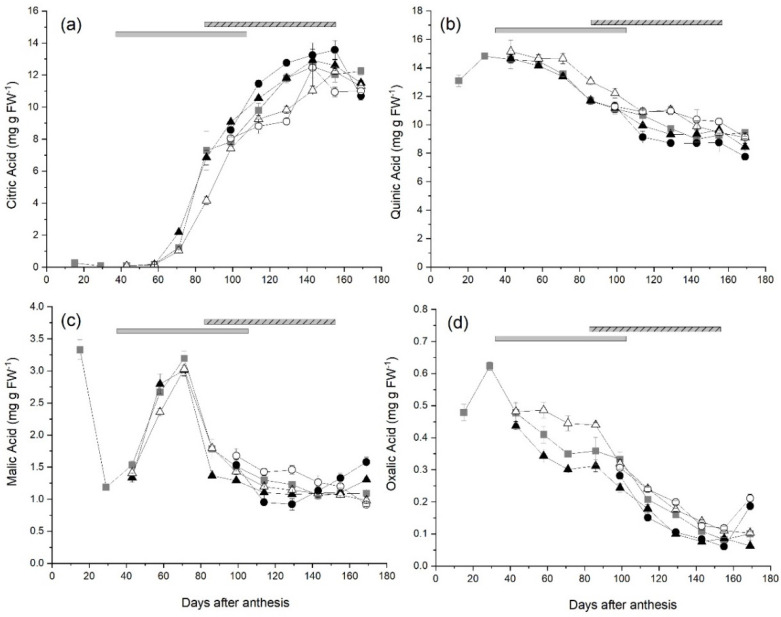
The effect of time of manipulation of carbohydrate supply together with phloem girdling on (**a**) citric acid (**b**) quinic acid (**c**) malic acid and (**d**) oxalic acid concentration of *Actinidia chinensis* var. *chinensis* ‘Zesy002’ kiwifruit from anthesis through to harvest. Data are shown for ungirdled control canes (■), early girdled canes with high (▲) or low (△) carbohydrate supply or late girdled canes with high (●) or low (○) carbohydrate supply. Early treatments were applied at 38 days after anthesis with the duration of the open girdle shown by the grey solid bar at the top of each panel. Late treatments were applied at 86 days after anthesis with the duration of the open girdle shown by the grey/black stripped bar at the top of each panel. High carbohydrate supply treatments had six leaves per fruit, while low carbohydrate supply treatments had one or two leaves per fruit. Data are means ± SEM, *n* = 5.

Citric acid concentrations in fruit from ungirdled control canes remained low until 58 DAA, increased rapidly reaching a peak of 12.5 mg g FW^−1^ at 143 DAA, and then plateaued around this concentration until harvest (Figure 3a). In fruit from canes receiving Early-High carbohydrate supply, citric acid concentrations were significantly higher than those in control fruit at 71 DAA. The citric acid concentrations in fruit with the Early-Low carbohydrate supply were significantly lower at 71 and 129 DAA and fruit from canes with the Late-Low carbohydrate supply at 129 DAA compared with those in control fruit. There were no significant differences between the concentrations of citric acid in fruit from ungirdled control canes or fruit from any other treatment at harvest (169 DAA).

The quinic acid concentrations in fruit from ungirdled control canes reached a peak of 14.8 mg g FW^−1^ at 29 DAA, and then steadily decreased to 9.44 mg g FW^−1^ at harvest (Figure 3b). The quinic acid concentration in fruit from the Late-High treatment was significantly lower than that in control fruit at harvest (169 DAA) but there were no effects of other treatments on quinic acid concentrations.

In fruit from control canes, the concentrations of malic acid decreased from 3.33 mg g FW^−1^ to 1.19 mg g FW^−1^ between 15 and 29 DAA, before increasing to a peak of 3.19 mg g FW^−1^ at 71 DAA (Figure 3c). Malic acid concentrations in control fruit declined rapidly to 86 DAA and then more gradually over the remainder of the season, reaching 1.09 g FW^−1^ at harvest (Figure 3c). In fruit from Early-High carbohydrate supply canes, the malic acid concentration was significantly less than that in control fruit at 86 DAA. However fruit from canes that received the Late-High carbohydrate supply had significantly lower malic acid concentrations at 114 and 129 DAA, but in contrast to the other treatments, concentrations in Late-High fruit then increased until harvest and were significantly higher (1.58 g FW^−1^) than those in control fruit at harvest. There were no significant effects of low carbohydrate supply treatments on malic acid concentrations in fruit during this study.

The mean concentration of available oxalic acid in fruit from ungirdled control canes increased to a peak of 0.623 mg g FW^−1^ at 29 DAA and then declined throughout the remainder of fruit development to 0.101 mg g FW^−1^ at harvest (Figure 3d). Fruit from the Early-High carbohydrate supply treatment had lower oxalic concentrations than control fruit at 129, 143 DAA and harvest (0.0627 mg g FW^−1^). While fruit from the Late-High supply also had lower oxalic acid concentrations than control fruit at 129 DAA, they also had significantly higher concentrations (0.186 mg g FW^−1^) than control fruit at harvest. Fruit from Late-Low carbohydrate supply canes had significantly higher concentrations of oxalic acid than fruit from control canes at both 155 DAA and harvest (0.211 mg g FW^−1^). There was no effect of treatments on fruit osmotic potential during fruit growth (Table S1 supplementary data).

### 2.4. Cytokinin Concentrations during Fruit Development

**Figure 4:**
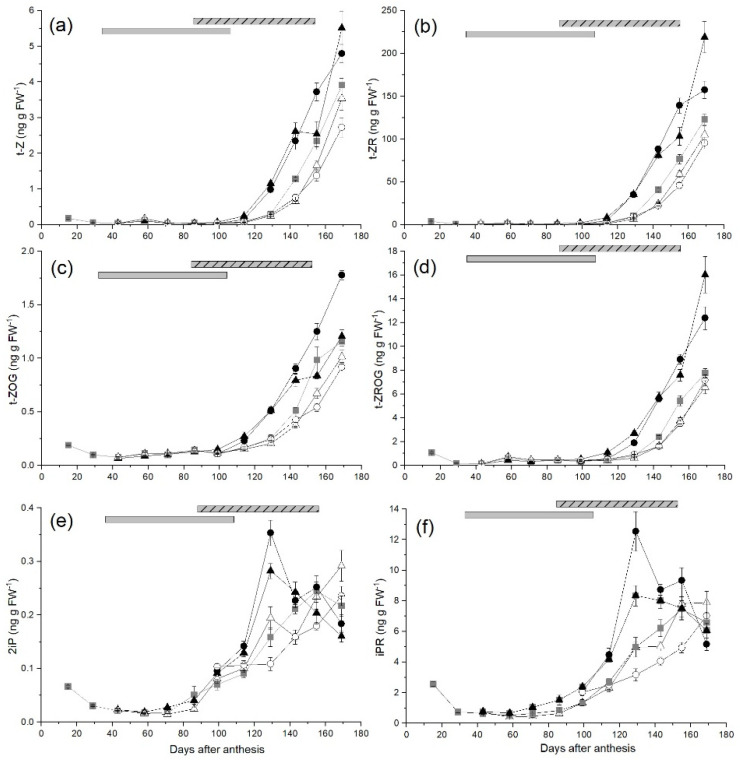
The effect of time of manipulation of carbohydrate supply together with phloem girdling on (**a**) *trans*-zeatin (t-Z) (**b**) *trans*-zeatin-O-glucoside (t-ZOG) (**c**) isopentenyl adenine (2iP) (**d**) isopentenyl adenine riboside (iPR), (**e**) *trans*-zeatin riboside (t-ZR) and (**f**) *trans*-zeatin riboside-O-glucoside (t-ZROG) concentration of *Actinidia chinensis* var. *chinensis* ‘Zesy002’, kiwifruit, from anthesis through to harvest. Data are shown for ungirdled control canes (■), early girdled canes with high (▲) or low (△) carbohydrate supply or late girdled canes with high (●) or low (○) carbohydrate supply. Early treatments were applied at 38 days after anthesis with the duration of the open girdle shown by the grey solid bar at the top of each panel. Late treatments were applied at 86 days after anthesis with the duration of the open girdle shown by the grey/black stripped bar at the top of each panel. High carbohydrate supply treatments had six leaves per fruit, while low carbohydrate supply treatments had one or two leaves per fruit. Data are means ± SEM, *n* = 5.

*trans*-Zeatin (*t*-Z) was the major active form of cytokinin in developing ‘Zesy002’ fruit followed by isopentenyl adenine (2iP), whose measured concentration was almost 16-fold lower than that of *t*-Z in control fruit at harvest (169 DAA) (Figure 4a,e, respectively). Concentrations of other active cytokinin forms, such as *cis*-zeatin (*c*-Z) and dihydrozeatin (DHZ), were below the detection level in this study.

The concentration of *t*-Z in ungirdled control fruit was low from the early stages of development (0.18 ng g^−1^ FW at 16 DAA) until 113 DAA but increased by more than 13-fold over 42 days from 127 DAA, reaching 3.91 ng g^−1^ FW at harvest (Figure 4a). *trans*-Zeatin riboside, the precursor of *t*-Z, was the most abundant cytokinin at harvest (122.9 ng g^−1^ FW; 84% of the total measured cytokinin pool), and followed a similar pattern to *t*-Z during fruit development with low concentrations until 113 DAA and then a rapid increase until harvest (Figure 4b). *trans*-Zeatin-*O*-glucoside (*t*-ZOG) and *trans*-zeatin riboside-*O*-glucoside (*t*-ZROG), the storage forms of *t*-Z and *t*-ZR respectively, were detected at low concentrations (between 0.08 to 1.10 ng g^−1^ FW) until around 113 DAA followed by rapid increases to 1.16 ng g^−1^ FW and 7.75 ng g^−1^ FW respectively at harvest (Figure 4c,d).

Several zeatin-type cytokinins, *t*-Z, *t*-ZR, *t*-ZOG and *t*-ZROG, responded similarly to changes in carbohydrate supply to fruit. In fruit from the Early-High treatments the concentrations of *t*-ZR at 86 DAA and *t*-Z at 99 DAA were significantly higher than those in control fruit. During the later stages of fruit development the concentrations of zeatin-type cytokinins were significantly higher in High carbohydrate supply fruit compared with Low carbohydrate supply fruit regardless of time of treatment application.

The concentration of 2iP in control fruit decreased from 0.07 ng g^−1^ FW at 16 DAA to 0.02 ng g^−1^ FW at 43 DAA and remained low until 71 DAA, then increased rapidly reaching a peak of 0.25 ng g^−1^ FW at 155 DAA, then decreased slightly to 0.22 ng g^−1^ FW at harvest (Figure 4e). Concentrations of isopentenyl adenine riboside (iPR) followed a similar pattern, decreasing from 2.56 ng g^−1^ FW at 16 DAA to 0.513 ng g^−1^ FW at 58 DAA prior to treatment application, remaining at low concentrations until 71 DAA, then increasing rapidly from 99 DAA to a peak of 7.47 ng g^−1^ FW at 155 DAA, before decreasing slightly to a final concentration of 6.59 ng g^−1^ FW at harvest (Figure 4f).

For the isopentenyl adenine-type cytokinins 2iP and IPR, there were also significant changes in response to changes in carbohydrate supply to fruit. The concentration of iPR in Early-High fruit was greater than that in control fruit at 71 and 86 DAA; and the concentration of 2iP at 86 DAA was significantly lower in Early Low fruit compared with those in control fruit. Peak concentrations of 2iP and iPR in fruit from both Early and Late High carbohydrate supply fruit were significantly higher than those in control fruit (44% higher for Late-High and 15% for Early-High) and the peak was reached ~26 days earlier at 129 DAA compared with 155 DAA in control fruit. In contrast, the concentrations of 2iP in Low-Early and Low-Late fruit and IPR (Low-Late fruit) were still increasing at harvest.

### 2.5. Maturation of Fruit

**Figure 5:**
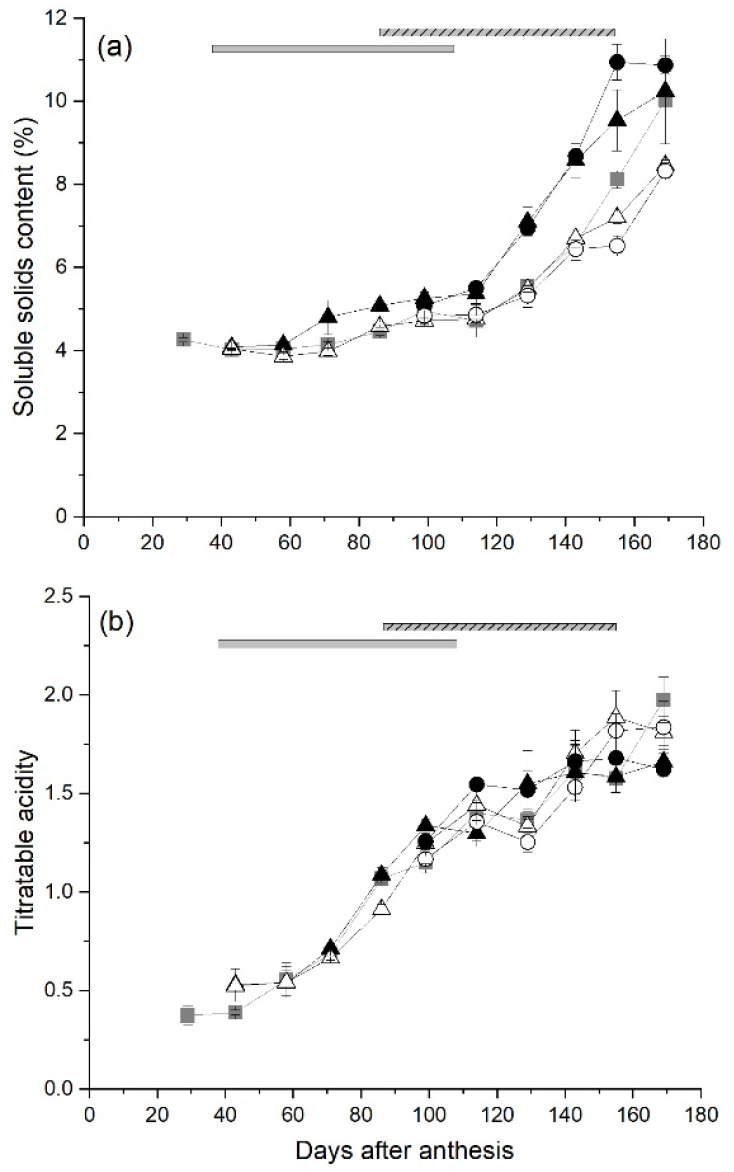
The effect of time of manipulation of carbohydrate supply together with phloem girdling on (**a**) soluble solids content and (**b**) titratable acidity of *Actinidia chinensis* var. *chinensis* ‘Zesy002’ kiwifruit during development. Data are shown for fruit from ungirdled control canes (■), Early girdled canes with High (▲) or Low (△) carbohydrate supply or Late girdled canes with High (●) or Low (○) carbohydrate supply. Early treatments were applied at 38 days after anthesis with the duration of the open girdle shown by the grey solid bar at the top of each panel. Late treatments were applied at 86 days after anthesis with the duration of the open girdle shown by the grey/black stripped bar at the top of each panel. High carbohydrate supply treatments had six leaves per fruit, while low carbohydrate supply treatments had one or two leaves per fruit. Data are means ± SEM, *n* = 5.

Fruit from control canes began to mature from 114 DAA, when soluble sugars started to accumulate (Figure 2 and Figure 5a). From 143 DAA until 155 DAA, fruit from canes that had a high carbohydrate supply (Early-High and Late-High) had significantly higher SSC than control fruit. In contrast, from 129–155 DAA fruit that had a low carbohydrate supply (Early-Low and Late-Low) had significantly lower SSC than fruit from canes with a high carbohydrate supply, but not control fruit (Figure 5a). However there were no significant effects of carbohydrate supply treatments on the titratable acidity (TA) of fruit during their development (Figure 5b).

Fruit maturation (fruit firmness and hue angle) were monitored from 135 DAA to 182 DAA. There were also differences in fruit firmness between control fruit and those from Late cane treatments. Fruit from canes with the Late-High carbohydrate supply were softer than fruit from control canes at 135 DAA (Late-High 8.33 kgf vs control 10.01 kgf) and at harvest 164 DAA (Late High 6.49 kgf vs control 8.08 kgf). However, fruit from canes with the Late-Low carbohydrate supply were significantly firmer than control fruit at 157 DAA and harvest (Late-Low 9.18 kgf vs control 8.08 kgf). Decreases in the hue angle of the fruit outer pericarp began earlier in fruit from canes with a High carbohydrate supply, with the hue angle of fruit from the Early-High treatment (105.3°) significantly lower than that of fruit from control canes (109.2°) at 135 DAA. Fruit from Late-High carbohydrate supply canes also had a significantly lower hue angle than control fruit at 135 DAA (104.9°) and 150 DAA (Late-High 102.7° vs control 105.0°). In contrast, the hue angle of the outer pericarp of fruit from both Low carbohydrate supply treatments was significantly higher than that of control fruit from 135–164 DAA (Early-Low 112.5–104.9°, Late- Low 112.3–106.6° vs control 109.2–102.7°).

### 2.6. Sensory Evaluation of Fruit

**Figure 6:**
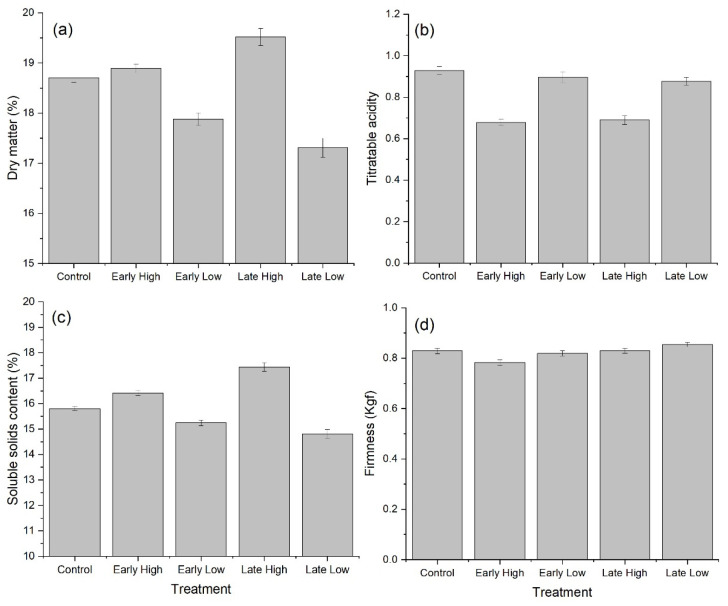
The effect of time of manipulation of carbohydrate supply together with phloem girdling on (**a**) dry matter (**b**) titratable acidity (**c**) soluble solids content and (**d**) firmness of *Actinidia chinensis* ‘Zesy002’ kiwifruit, in fruit used in the consumer sensory panel. Early girdled canes with High or Low carbohydrate supply or Late girdled canes with High or Low carbohydrate supply. Early treatments were applied at 38 days after anthesis and Late treatments were applied at 86 days after anthesis. Fruit were stored for 10 weeks at 2 °C for around 10 weeks and then ripened at 20 °C for up to 7 days before sensory analyses. High carbohydrate supply treatments had six leaves per fruit, while low carbohydrate supply treatments had one or two leaves per fruit. Data are means ± SEM, *n* = 75.

The average DM of ripe fruit from ungirdled control canes prepared for sensory analysis after cold storage and ambient ripening was 18.7%. This was significantly lower than that of fruit from the Late-High carbohydrate supply (19.5%), but significantly higher than that of fruit from either the Early-Low (17.9%) or Late-Low (17.3%) carbohydrate supply treatments (Figure 6a). The differences in fruit SSC followed a similar pattern to DM of fruit (Figure 6c). Fruit from both the Early- and Late-High carbohydrate supply treatments had significantly higher SSC than control fruit and fruit from canes with Early-Low or Late-Low carbohydrate supply treatments had significantly lower SSC than control fruit. The TA of fruit from either Early- or Late-High carbohydrate supply canes was significantly lower than that in fruit from control canes (Figure 6b). Therefore the SSC/TA ratio in fruit from Early-High (25.5) and Late-High (27.9) carbohydrate supply canes were significantly higher than those from control (18.6), Early-Low (18.6) and Late-Low (17.6) carbohydrate supply canes. Fruit selected for presentation to the sensory panel were matched for firmness and therefore had similar firmness to control fruit (0.83 kgf) with the exception of fruit from Early-High carbohydrate supply canes that were slightly, but significantly softer than control fruit at 0.78 kgf (Figure 6d).

Evaluation of perceived fruit characteristics, including flavour (tropical, metallic, lemon/lime, grassy/green), taste (sour/acidic, sweet) and texture (juicy, crunchy, soft, mushy-melting/smooth), and general quality (bland, fresh, off flavour, under-ripe and over-ripe), were made by consumers. Only the citation frequencies (percentage of consumers selecting a term) for sour/acidic, sweet and under-ripe showed significant treatment effects. These data are presented in Table 1 and reflect measurements in Figure 6 Fruit from ungirdled control canes were described by significantly more consumers as sour/acidic compared with fruit from Early-High carbohydrate supply canes. More consumers described fruit from ungirdled control canes as sweet compared with fruit from canes with either the Early-Low or Late-Low carbohydrate supply. In terms of perceived ripeness, significantly more consumers described fruit from canes with the Late-Low carbohydrate supply as under-ripe than those from canes with the Early-High carbohydrate supply treatment.

## 3. Discussion

In the current study we investigated how manipulating the supply of carbohydrate from source leaves to fruit sinks at different stages of development influenced the growth, sugar, acid and cytokinin metabolism and maturation of ‘Zesy002’ fruit. Increasing carbohydrate supply to fruit during early development had a major impact on fruit growth rates, while increased carbohydrate supply later in fruit development had more effect on fruit composition through increased SSC compared with control fruit. Fruit maturation was advanced by increasing carbohydrate supply at either stage of fruit sink development. In contrast, reducing the supply of carbohydrate to fruit slowed dry weight accumulation, changed fruit composition through reduced soluble sugar concentrations and slowed fruit maturation processes. Changes in fruit maturation in response to changes in carbohydrate supply appeared to be associated with effects on fruit cytokinin metabolism. Carbohydrate supply to fruit sinks also influenced consumer perceptions of the sweetness, acidity and ripeness of the fruit.

### 3.1. The Effect of Carbohydrate Source Manipulation on Fruit Growth

The growth of *Actinidia* spp. fruit generally occurs in two stages: an initial rapid period of growth followed by a second slower period of growth [19,20]. The pattern of rapid early fresh weight increase of ‘Zesy002’ was similar to that in a wide range of *Actinidia* spp. including *A. chinensis* var. *deliciosa* [19], *A. chinensis* var. *chinensis* [20] and *A. arguta* [30]. However in the second slower phase of fruit development, fruit growth of ‘Zesy002’ continued at a relatively rapid rate until 143 DAA and this higher rate of later growth may explain why this genotype can produce large fruit [2]. Increasing the carbohydrate source, either during the initial stage of fruit growth or the second stage of development, stimulated fruit sink growth rates. This resulted in significant increases in fruit size at harvest compared with control fruit. Previous studies have shown that growth of fruit from *Actinidia* spp. can be readily influenced by environmental factors (water and temperature) [38,50] and management practices (pollination, [51] thinning [52], girdling [33] and application of growth regulators [34,40]) that influence carbohydrate supply to fruit sinks during the first stage of growth. However no other *Actinidia* spp. fruit have shown such high rates of growth during the second stage of growth or such large responses to carbohydrate supply.

Early fruit growth rates in *Actinidia* spp. have been attributed to osmotic potential from the combined accumulation of glucose [21,22] and quinic acid [18,24] as well as hormonal stimulation of growth by cytokinin [42] during cell division in fruit. In the current study there was a small early peak in glucose concentrations in ‘Zesy002’ fruit around 29 DAA, before treatments were applied, that was similar to that shown in *A. deliciosa* var. *deliciosa* genotypes [21] and ‘Hort16A’ [18]. The small peak in quinic acid in ‘Zesy002’ fruit at 29 DAA was similar to that in *A. arguta* but was smaller and earlier than that in *A. deliciosa* var. *deliciosa*, *A. chinensis* var. *chinensis* and *A. eriantha* [53]. Cytokinin concentrations were also elevated in ‘Zesy002’ fruit in this study during early fruit development (15–29 DAA) suggesting that cell division in this genotype occurred during the very early stages of kiwifruit growth [54] before treatments were applied. Therefore increases in fruit growth through high carbohydrate source supply to fruit sinks in this study were likely due to increased cell expansion [55]. A previous study showed that the increased rate of fruit growth resulting from exogenous application of cytokinins was due to increased osmotic potential and hence water uptake by cells in *Actinidia* spp. [40]. However, in the current study there were no significant changes in osmotic potential within fruit that could be related to the measured increase in fruit growth rates. Therefore it is possible that carbohydrate supply has influenced fruit growth via cellular processes other than osmotic potential and cytokinin concentration, and that production of expansins [42] and the supply of essential metabolites for growth is carbohydrate limited.

The DW increase of control ‘Zesy002’ fruit was linear between 40 and 126 DAA in this study and similar to linear increases in DW between 28 and 140 DAA in ‘Hort16A’ fruit [18], although the rate of DW increase in ‘Zesy002’ was higher than that in ‘Hort16A’ [18]. Effects of modifying carbohydrate source supply on fruit sink DW accumulation were similar to those for FW, supporting a direct effect of treatments on cellular development and metabolite accumulation. Changes in fruit DM reflected the relationship between DW and FW growth, with a sharp decline after anthesis as the FW of fruit increased rapidly, a pattern shown in other *Actinidia* genotypes [18,38]. However, there was a much longer period when fruit DM remained at a minimum, before rapid DW accumulation began, compared with that reported in other *Actinidia* genotypes. Modifying carbohydrate source supply to ‘Zesy002’ fruit in this study, particularly during the second stage of fruit sink development, significantly increased the DW accumulation and hence DM accumulation in fruit. Effects of carbohydrate supply treatments on fruit sink DM accumulation were similar to those reported in earlier studies of *Actinidia* spp. where effects of temperature [38], girdling [45,56], crop load [35] and genotype [4] were studied. However previous studies have not shown such an extended period of balanced FW and DW accumulation when DM is at a minimum.

### 3.2. Fruit Metabolite Accumulation Is Affected by Carbohydrate Supply to Fruit

Starch, soluble sugars and acid accumulation in ‘Zesy002’ fruit all increased rapidly during the second stage of fruit development in this study. Rapid accumulation of starch in fruit began at the end of the first stage of fruit growth (from 58 DAA) and starch concentrations peaked at 143 DAA in control fruit. This is similar to previous studies where starch accumulation in *A. deliciosa* var. *deliciosa* and *A. chinensis* var. *chinensis* fruit peaked at 160 DAA and fruit had a similar development period to ‘Zesy002’ fruit, but differs from that in *A. arguta* fruit (50–80 DAA) which have a much shorter period of fruit development [22]. During the later stages of ‘Zesy002’ fruit development, when fruit began to mature, there was an increase in fruit soluble sugars as net starch degradation began, this is also typical of many *Actinidia* spp. genotypes [18,21,22].

Increasing the source of carbohydrate to fruit sinks at either stage of development advanced and increased the peak starch concentration in fruit and hence increased the accumulation of soluble sugars in fruit during maturation. In contrast, decreasing the carbohydrate supply to fruit at either stage of development delayed and reduced starch accumulation as well as reducing the subsequent accumulation of soluble sugars in fruit. A previous study has shown that increased starch accumulation in contrasting genotypes was related to increased ADP-glucose pyrophosphorylase (AGPase) and sucrose phosphate synthase (SPS) activity during rapid cell expansion of fruit [21]. These substantial treatment effects on starch and subsequent soluble sugar accumulation were reflected in fruit SSC and consumer responses for ripe fruit. Consumers were less likely to describe fruit from either early or late low carbohydrate supply treatments as sweet compared with control fruit. Therefore manipulating carbohydrate supply to fruit at either stage of development is a useful technique to make a large impact on final fruit flavor and consumer acceptability.

Quinic and citric acid were the main acids found in ‘Zesy002’ fruit, but showed contrasting patterns of accumulation. Quinic acid peaked at 29 DAA and then concentrations declined throughout the remainder of fruit growth. This was similar to the pattern for quinic acid in *A. arguta* fruit but contrasts with the higher and later peaks of quinic acid measured in fruit during the development of ‘Hort16A’, *A. deliciosa* var. *deliciosa*, *A. chinensis* var. *chinensis* and *A. eriantha* genotypes [18,57,58]. Citric acid accumulated in ‘Zesy002’ fruit during the second phase of growth and fruit maturation. This was similar to the accumulation pattern for citric acid previously shown in ‘Hort16A’ [18] and *A. eriantha* [58] fruit but contrasts with that in other *Actinidia* spp. [53]. Oxalic acid was present in ‘Zesy002’ fruit at much lower concentrations than citric and quinic acids, but followed a similar development pattern to quinic acid. Accumulation of oxalic acid in fruit was similar to that reported in an *A*. *eriantha* genotype [58]. Concentrations of malic acid initially declined, increased to a second peak at the end of rapid fruit growth, and then declined rapidly at the beginning of the second period of slower fruit growth. This is similar to the patterns reported for malic acid in ‘Hayward’ and ‘Hort16A’ fruit [18,53].

Accumulation of citric acid in fruit was affected during Early manipulations of carbohydrate supply to fruit sinks, however there were no effects of treatments on citric acid concentrations in fruit at harvest. Fruit from Late-High carbohydrate supply canes had lower quinic and higher oxalic and malic acid concentrations at harvest than fruit from control canes, while fruit from Late-Low supply canes also had higher oxalic concentrations at harvest. Once ‘Zesy002’ fruit had ripened, the titratable acidity of fruit juice, which has been shown to better reflect perceived acidity than fruit pulp [59], was lower in fruit from canes with a High carbohydrate supply suggesting that fruit maturity was more advanced. Consumers described these fruits as less sour/acidic than fruits from other treatments as they had a much higher SSC/TA, reinforcing the impact of carbohydrate manipulations on consumer responses.

### 3.3. Effects of Carbohydrate Supply on Fruit Maturation

In ‘Zesy002’ fruit SSC began to accumulate rapidly from 129 DAA, although net starch loss and the resulting accumulation of sucrose in fruit did not occur until 143 DAA when fruit growth ceased. It has been suggested that the delay in the cessation of growth and net starch breakdown until after the increase in SSC in ‘Hort16A’ kiwifruit was due to continued carbohydrate import into fruit even after starch accumulation ceases [60]. Fruit maturation was affected by altering the supply of source carbohydrate to fruit sinks during their development. Cessation of fruit growth, net starch loss, accumulation of soluble sugars and development of flesh colour began earlier in fruit that had a high carbohydrate source supply. In contrast, fruit sink growth continued for longer and accumulation of soluble sugars and colour development were delayed in fruit with a low carbohydrate supply. Changes in the carbohydrate supply to fruit during the late stage of fruit development influenced fruit softening; Late-High carbohydrate supply fruit softened earlier and Late-Low fruit softened later than fruit from Early treatment and control canes. Previous studies have shown that modifying the carbohydrate source supply to fruit sinks by treatments such as crop load and phloem girdling can modify fruit maturation [35,49]. Although fruit in this study were matched for maturity, fewer consumers described fruit from the Early-High carbohydrate supply treatment as under-ripe than fruit from the Late-Low carbohydrate supply. This is likely due to the differences in the SSC/TA ratio but could also be influenced by effects on volatile profiles in fruit as these factors can influence consumer perception of ripeness and sourness [28]. Therefore altering fruit carbohydrate supply, by for example thinning or more focused use of girdling, could be a useful techniques to alter the timing of fruit maturation for harvest and market supply.

Changes in cytokinin concentrations in ‘Zesy002’ fruit appeared to be associated with changes in fruit maturation as previously shown in other green- and yellow-fleshed *Actinidia* genotypes [61]. Concentrations of zeatin- and isopentyladenine-type cytokinins began to increase at around 120 DAA. Changes in concentrations were advanced and increased in ‘Zesy002’ fruit that had received a high carbohydrate supply. In contrast, increases in cytokinin concentrations in fruit with low carbohydrate supply tended to be lower than those in control fruit. Previous studies of a red-fleshed *A. chinensis* var. *chinensis* genotype showed a large effect of carbohydrate starvation on cytokinin concentrations but not on other hormones in fruit [42]. That study showed that cytokinin synthesis was induced by sugar with a possible relationship between trehalose-6-phosphate and cytokinin signaling [42]. The results from the current study also support a link between cytokinins and sugars in *Actinidia* spp. fruit and a key role of cytokinin in fruit maturation.

## 4. Materials and Methods

### 4.1. Plant Material

Three-year-old ‘Zesy002’ vines grafted onto mature *A. chinensis* (Planch.) var. *deliciosa* ‘Bruno’ seedling rootstock growing in an orchard in Karapiro, New Zealand (37°56′13′′ S, 175°32′40′′ E) were used for this study. Vines were trained on a pergola system and managed according to standard organic management techniques. All vines were thinned to an initial L:F ratio of three at fruit set. The date of anthesis (defined as when 50% of flowers had opened) was 9 November 2015. Treatment and sampling dates were referred to as DAA.

### 4.2. Experimental Design

The effects of carbohydrate source supply to fruit were investigated using single one-year-old canes as experimental units. Ten uniform canes were selected on each of the 27 vines, giving 270 canes in total with a L:F of 3. Each treatment was randomly applied to two canes on each vine. Treatments included Low (one to two leaves per fruit) or High (six leaves per fruit) carbohydrate supply, that were applied to canes together with a basal phloem girdle either Early (38 DAA) or Late (86 DAA) in fruit development. A 5 mm basal girdle was applied between the base and the first shoot of the treated one-year-old canes, using girdling pliers (Vaca, Wenatchee, Washington, DC, USA). Girdles were applied once at the start of the treatment and healed after approximately 70 days (108 DAA for Early treatments and 156 DAA for Late treatments). The L:F ratios of treated canes were adjusted on the same day that girdles were applied by removing either fully formed leaves or fruit as necessary and these were maintained throughout the experiment by removing any new leaves. These treatments (Early-High, Early-Low, Late-High and Late-Low) were compared with control canes that were ungirdled and had a moderate carbohydrate supply (three leaves per fruit).

### 4.3. Fruit Measurements

Fruit were sampled at fortnightly intervals, from 15 DAA until 169 DAA. On the first two sampling dates (15 and 29 DAA), before treatments were applied, fruit were only sampled from control canes. Thereafter fruit were sampled from control and Early treatments from 43 DAA and all treatments from 99 DAA. At each sampling date five fruit were sampled from each of five randomly selected canes per treatment. Canes were only sampled once during the experiment.

At each sampling date the fresh weight of each harvested fruit was recorded. Each fruit was cut in half longitudinally and a subsample was cut into small pieces and then bulked across the five fruit sampled per cane. The bulked sample was then divided into two; one sample for DM and the other for chemical analysis. The sample for DM measurement was weighed and both samples were immediately frozen in liquid nitrogen and then stored at −80 °C for further analysis. The sample for DM measurement was freeze dried, reweighed and the FW and DW of the sample were used to calculate the sample DM. This was used with the FW to calculate the average DW of the whole fruit sampled from each cane.

### 4.4. Soluble Carbohydrates, Starch, Organic Acid and Cytokinin Analyses

Samples for chemical analysis were ground using a liquid nitrogen chilled IKA™ A11 basic analytical mill (IKA-Works Asia, Kuala Lumpur, Malaysia) and the powder was stored at −80 °C for analysis. A subsample was then defrosted, spun at 4000 rpm and the supernatant was decanted for TA and SSC assessments. TA analyses were carried out on a 50 μL subsample of the juice supernatant placed into a 96 well plate and 20 μL of 0.1% m-cresol purple dissolved in 20% ethanol was then added to each well. The plate was then shaken on a vibrating mixer (IKA Vibrax-VXR with a Janke and Kunkle Typ VX1 plate holding attachment, (IKA-Works Asia, Kuala Lumpur, Malaysia) and vibrated at 600 rpm. Titration of 0.01 M NaOH using a Rainin EDP Plus electronic pipette (Mettler Toledo, Columbus, OH, USA) was carried out to the point at which a stable purple colour formed. Standard curves were used to calculate citric acid equivalents. A subsample of the supernatant was used to determine SSC with a digital refractometer (Atago, Tokyo, Japan).

Soluble carbohydrates were extracted from 0.2 g FW samples in 80% (*v/v*) ethanol. The soluble carbohydrates (glucose, fructose, sucrose, *myo*-inositol and galactose) contents were analysed by ion chromatography on a DIONEX ICS-5000 Reagent-Free™ IC (RFIC™) system (Thermo Scientific, San Jose, CA, USA) equipped with a CarboPac PA20 column [21]. Starch content was analysed by an enzymatic digestion of the insoluble residue from the ethanolic extraction, and colorimetric measurement of glucose [55].

Organic acid concentrations were measured using a Dionex UltiMate 3000 Series UHPLC (ThermoFisher Scientific, Waltham, MA, USA) with photodiode array (PDA) detection at 210 and 220 nm. Compound separation was achieved isocratically with 0.08% phosphoric acid at 1 mL/min using a Synergi 4µ hydro-RP 80A column, 4.6 × 250 mm (Phenomenex, Torrance, CA, USA), maintained at 30 °C. Sample injection volume was 10 µL. Ground tissue samples (1 g) were extracted twice with chilled 0.5% *meta*-phosphoric acid, and the supernatants combined and centrifuged prior to injection. The concentration of soluble carbohydrates and organic acids were then used to calculate total fruit osmotic pressure [40].

Cytokinins were extracted, fractionated and then quantified by liquid chromatography-mass spectrometry [42] with the following modifications. Cytokinins were separated on a Poroshell 120 SB-C18 2.7 µm, 2.1 × 150 mm ID column (Agilent, Santa Clara, CA, USA) maintained at 80 °C. Solvents were (A) water with 0.1% formic acid and (B) acetonitrile with 0.1% formic acid and a flow rate of 600 μL min^−1^. The initial mobile phase, 100% A was ramped linearly to 3% B at 9 min, then to 10% B at 15 min and 100% B at 22 min and holding at 100% B for 1 min before resetting to the original conditions. Injection size was 10 µL.

### 4.5. Fruit Maturity Assessments, Harvest and Fruit Ripening

At 135 DAA and then weekly from 150 DAA to 182 DAA, four fruit from each of five canes per treatment were sampled to monitor fruit maturity. Flesh firmness was measured using a Fruit Texture Analyser (Güss model GS14, Strand, South Africa) with a 7.9 mm probe, at two adjacent sites at the equator of fruit [47]. The hue of the outer pericarp of fruit was measured at the equator of the fruit using a Minolta chromameter CR300 (Minolta, Ramsey, NJ, USA) using the method of Montefiori, et al. [62].

Fruit were considered mature and ready to be harvested when the average SSC was greater than 7% and the hue angle of the outer pericarp was less than 104°. Fruit met these maturity criteria at 170 DAA for control and high carbohydrate supply treatments and 184 DAA for low carbohydrate supply treatments. From each treatment, 150 fruit were harvested from canes that had not previously been sampled. The fruit were then stored at 2 °C for around 10 weeks and then ripened at 20 °C for up to 7 days until they reached eating-ripe firmness.

### 4.6. Consumer Evaluation

The consumer preference experiment was carried out over four days and fruit were prepared each morning. Half of each fruit was used to measure firmness, colour and DM of the fruit as previously described and SSC using a hand-held refractometer (0–20%, Atago). The second half of each fruit was used for the sensory experiment. In total 78 untrained participants were surveyed, with 18–20 participants per day. The panel comprised participants aged 18–84 years, (59% aged 18–30 years, 16% in the 31–45 year age group and 24% 46 years or older), with an approximately even ratio of males and females (53% and 47% respectively). Of these, 28% reported eating kiwifruit commonly (at least once a week when in season), 32% ate kiwifruit occasionally (one to three times a month), and 40% reported eating kiwifruit rarely (once every few months to never). Of the participants, 48% reported to like gold kiwifruit very much or extremely, 32% moderately liked gold kiwifruit, 1% neither liked nor disliked gold kiwifruit, and 5% disliked gold kiwifruit.

Ethical approval was gained from the University of Waikato Human Research Ethics Committee, and the project adhered to the University of Waikato Human Research Ethics Regulations 2008 and the ethical guidelines of the New Zealand Association for Research in Education.

For sensory analysis each fruit sample was washed and sliced into wedges. Each participant evaluated one sample from each of the five treatments, and samples were presented at room temperature, in plastic custard cups with random three-digit codes corresponding to the different treatments. These were served, one at a time, to participants to taste and fill out a short questionnaire about perceived taste, flavour and texture. Samples were presented in a randomised order based on a complete block design, to minimise first order and carry-over effects. Participants were provided water and a plain water cracker (Arnott’s Original Water Crackers, Arnott’s Biscuits Ltd., Homebush, NSW, Australia) between each sample to cleanse their palate.

“Check all that apply” (CATA) questions allow consumers to rapidly perform sensory product characterisation by selecting from a pre-defined list of sensory attributes all the terms that apply to the focal product. The list of terms used in this study included a range of flavour (tropical, metallic, lemon/lime, grassy/green), taste (sour/acidic, sweet), texture (juicy, crunchy, soft, melting/smooth, mushy) and general fruit quality terms (bland, fresh, off-flavour, over-ripe, under-ripe). The order of these attributes were randomised between surveys as to avoid response bias, based on a Williams Latin square design.

### 4.7. Statistical Analysis

Data were checked for normality, log transformed where necessary and statistically analysed using GenStat 17th edition for Windows (VSN International Ltd., Hemel Hempstead, Hertfordshire, UK). The effects of carbohydrate supply and fruit age on fruit growth and fruit starch, sugar, acid and cytokinin composition were determined on mean data per cane unit using a two-way ANOVA with treatment and DAA as factors. A Bonferroni multiple comparison test was used to assess the significance between means at *p* < 0.05 and all significant differences quoted in the text are at this value. A correspondence analysis was carried out on the CATA data, with Cochran’s Q test used to identify whether the citation frequency of each term differed between treatments (*p* < 0.05). Responses from three respondents were excluded in the analysis because they did not have a complete and balanced set of observations.

## 5. Conclusions

The growth and development of ‘Zesy002’ fruit sinks was highly responsive to changes in carbohydrate source supply. Increasing the carbohydrate source supply to ‘Zesy002’ fruit sinks during fruit development increased fruit growth by up to 38% during early development and increased the accumulation of soluble sugars by up to 79% during late development. In contrast reducing carbohydrate source supply decreased fruit sink soluble sugar accumulation by up to 36%. Advances in fruit maturation through a high carbohydrate supply and delays with a low supply were linked to the accumulation of cytokinin compounds in fruit, suggesting an association between sugar and cytokinins. Increasing the supply of source carbohydrate to fruit either early (38 DAA) or late (86 DAA) in fruit development provides a useful tool to increase fruit growth, flavour composition and maturation of fruit. Changes in fruit properties through altering their carbohydrate source supply affected consumer perceptions of fruit maturity and sugar acid balance.

## Figures and Tables

**Table 1:** Citation frequencies (% of consumers) for terms used to describe the sensory attributes for *Actinidia chinensis var. chinensis* ‘Zesy002’, kiwifruit from five treatments receiving varying carbohydrate supplies. *n* = 75 consumers. Statistical significance (*p* < 0.05) is shown for descriptors using different letters following citation frequencies.

Descriptors	Control	Early High	Early Low	Late High	Late Low
Bland	18.7a	13.3a	13.3a	10.7a	17.3a
Crunchy	4a	4a	5.3a	4a	2.7a
Fresh	36a	29.3a	36a	36a	36a
Grassy/green	2.7a	4a	6.7a	5.3a	8a
Juicy	49.3a	54.7a	49.3a	60a	50.7a
Lemon/lime	20a	9.3a	22.7a	20a	24a
Melting/smooth	44a	44a	37.3a	48a	41.3a
Metallic	2.7a	2.7a	2.7a	2.7a	4a
Mushy	20a	34.7a	24a	25.3a	20a
Off-flavour	1.3a	4a	2.7a	4a	10.7a
Over-ripe	13.3a	21.3a	13.3a	10.7a	10.7a
Soft	69.3a	73.3a	62.7a	72a	61.3a
Sour/acidic	38.7ab	20.0c	44.0a	26.7bc	44.0a
Sweet	57.3a	66.7a	41.3b	72.0a	40.0b
Tropical	21.3a	18.7a	18.7a	20a	20a
Under-ripe	6.7ab	1.3b	4.0ab	2.7a	12.0a

## Data Availability

The data presented in this study are available on request from the corresponding author (A.R.). The data are not publicly available due to privacy reasons.

## Supplementary Materials

The following are available online at https://www.mdpi.com/article/10.3390/plants10071328/s1, Table S1. The effect of time of manipulation of carbohydrate supply and phloem girdling on total fruit osmotic potential during fruit growth. Early treatments were applied at 38 days after anthesis, while Late treatments were applied at 86 days after anthesis. Data are means ± SEM, *n* = 5.
