# Supplementary material for: Modifying Carbohydrate Supply to Fruit during Development Changes the Composition and Flavour of Actinidia chinensis var. chinensis ‘Zesy002’ Kiwifruit
Source: Plants (Basel). 2021 Jun 29;10(7):1328. doi: 10.3390/plants10071328 (PMC8309063; doi:10.3390/plants10071328)
Supplement: Supplementary file 1 [file plants-10-01328-s001.zip › plants-1270558-supplementary.pdf]

Table S1. The effect of time of manipulation of carbohydrate supply and phloem girdling on total fruit osmotic potential during fruit growth. Early treatments were applied at 38 days after anthesis, while Late treatments were applied at 86 days after anthesis. Data are means ± SEM, n = 5.

Days after anthesis	Control	Early High	Early Low	Late High	Late Low
**43**	0.490	0.512	0.462	_	_
**58**	0.490	0.500	0.495	_	_
**71**	0.492	0.553	0.479	_	_
**86**	0.465	0.512	0.520	_	_
**99**	0.571	0.605	0.562	0.568	0.562
**114**	0.576	0.633	0.565	0.607	0.459
**129**	0.603	0.795	0.601	0.785	0.549
**143**	0.722	0.892	0.657	0.948	0.642
